# Clinical Study in Typhoid Fever*Read before the Maine Medical Association, June 6th, 1895.

**Published:** 1895-11

**Authors:** D. E. Parson

**Affiliations:** Oakland, Me.


### A CLINICAL STUDY IN TYPHOID FEVER.*

BY D. E. PARSON, M. D., OAKLAND, ME.

In presenting these clinical notes on typhoid fever, I do not expect to add an iota to your knowledge of the disease, or present any facts new in its treatment. My motive is to record my observations and call your attention to results which I have obtained in a country practice extending over a period of twenty-nine years, through means long known to every practitioner of medicine.

Kindly bear with me for calling your attention anew to points in regard to cause of the disease, seemingly at variance with the present generally accepted teachings of modern investigators, which I am led to do because I am unable to reconcile these teachings with my own personal observations. For twenty-five years the theory has been advocated, and is now generally accepted, that every case of typhoid fever is the indirect product of a previous case; that the disease is not directly contagious through the atmosphere, but that the germ which produces the disease, usually taken into the system through the alimentary canal, is the product of the decomposition of the discharges from a typhoid fever patient. There is abundant evidence to prove that one case of typhoid fever has sprung from another; that epidemics have been produced by single cases. There is, however, a class of cases constantly met with in a country practice, whose origin will not admit of such an explanation. Let me present to you these illustrative cases, such as the country physician each year, in his practice, meets in the cottage of the village, the home of the farmer or cabin of the pioneer. W. C. and W. B., living in a neighborhood where there had not been another case for twenty years, worked together through the haying season, ate of the same food, and drank of the same water from the same well. August 21st and 23d, respectively, I was called to them, sick with typhoid fever; on investigation, I found that the sink drain had discharged its contents, by following the course of a wall, into the well from which they had obtained their

*Read before the Maine Medical Association, June 6th, 1895.

supply of water. If you reply that these were not true cases of typhoid fever, but cases of a filth disease, as there is no evidence that the typhoid bacillus was present in the water, my answer would be, we have no typhoid fever in the country, it is all a filth disease. Whether the typhoid bacillus is necessary or not to produce the fever, I do not know; that it will produce the fever when introduced into the human system is not known, but if the bacillus necessarily enters the body of every typhoid fever patient in the manner now taught, I am satisfied that its origin is not always from a previous case. I will not weary you with the details of other cases to illustrate this point, as my object is to consider the treatment and not the ætiology of the disease.

There are no symptoms, subjective or objective, in many cases during the first few days, whereby typhoid fever can be diagnosticated. Including those cases which are often called slow or continued fever, many cases run their courses without developing any positve symptoms of the disease. The nose bleed is wanting, the rose spots do not appear, there is no diarrhœa, tympanites come not to our aid; every sign may be absent, except that the patient is ill and has fever. The country doctor must make his diagnosis within a few days, and is often obliged to form his opinion by the exclusion of other diseases, instead of any positive signs of this disease. In my experience, the most perplexing and long continued uncertainty has been caused by complications from other diseases, especially an early and persistent bronchitis. The complications and sequelæ which I have observed have not been frequent or numerous. After convalescence had been established, I did not find a single case which I was able to consider a relapse; cases occasionally occur where, at the end of the second and some a week later, the fever has abated, and other signs indicated that the disease would soon terminate, when instead of such a course there has been a renewal of the fever and a continuation of the disease a week or two longer, caused undoubtedly by the intestinal lesions. The complications, in my cases, which do not frequently occur, have been inflammation of both parotid glands, ending in resolution; jaundice after convalescences was established; acute monomania in the fourth week after the temperature and pulse indicated the termination of the fever; thrombosis of the crural veins, multiple abscess of the cellular tissues of the trunk; pneumonia, one case of which commencing quite early in the disease, resulted in abscesses of the lung, which discharged through the bronchi; one case of general peritonitis, commencing after the third week, when the patient was apparently convalescent; one case of hemorrhage from the bladder in the second week; one case of abortion at three and a half months, during the third week of the fever; and one case in which a profuse rash covered the whole trunk, limbs, and face, in the fourth week, following an extensive sudamina.

It is, I think, conceded that death generally results from three distinct conditions:

First. The destructive changes in the tissues and organs, incident to the high grade of the fever.

Second. Asthenia from the long continued fever and inadequate nutrition.

Third. Intestinal hemorrhage and perforation.

If these conditions can be successfully controlled and complications avoided, and our patients are not previously afflicted with some organic disease, then our treatment will prove uniformly successful.

The first condition, at the beginning of my practice had to be met with remedies then at our command which now seem totally inadequate. The days of bloodletting, fortunately, having passed, we had, with which to reduce the temperature, cold water, which then and now, in a country practice, without adequate means and with inexperienced attendants can only be successfully used for a beverage and for the sponge bath; refrigerant diaphoretics, viz.: citrate of potassa, acetate of ammonia, nitrate of potassa, and sweet spirits of nitre, which were as inefficient as they were harmless; arterial and nervous sedatives, of which the tincture of aconite and veratrum viride were the most efficient, as well as the most dangerous.

Mrs. M., a widow, aged 33, had been ill ten days with a moderately severe fever, had been taking two drops tincture of aconite root every four hours for nearly a week, without any perceptible effect on her temperature or circulation, when I increased the dose to three drops every four hours. Early the next morning I was summoned in haste with the statement that the patient was much worse. I took with me an older and experienced physician, with whom I was then associated in practice, and hastened to the bedside of the sufferer; found her semiunconcious, unable to speak, bathed in a profuse, cold perspiration, pulse and temperature—clinical thermometers were not in use—sub-normal.

My colleague diagnosticated apoplexy, advised a blister to the spine, which the nurse, who made a second application of the cantharidal visicant, painted all over her back. Concurring with my counsel, not, however expressing my opinion in regard to the aconite, which he knew had been used, I administered a liberal supply of brandy and other stimulants and had the satisfaction of seeing my patient restored from impending death. The fever resumed its normal course in a few days; and in spite of the aconite and terribly blistered back, the patient, in the fourth week, entered into a satisfactory convalescence.

Among my early instructors, Dr. Shattuck impressed upon me the importance of "appealing to the emunctories" in typhoid fever, as it is one of the "zymotic diseases," and that impression produced a lasting effect upon my mind. Since the days of refrigerant diaphoretics, aconite and veratrum viride went by with me and the modern antipyretics have come into use, I have administered them for the double purpose—their diaphoretic action and antipyretic effect. The impression remaining with me, that the poison, in part, is eliminated through the pores of the skin, I have used them when not indicated on account of the high grade of the fever, for the purpose of eliminating the poisonous products of the disease by means of the perspiration, also for reduction of the temperature, when above 102 1-2; and none has served me better than phenacetine, which, when given in from five to ten-grain doses, has usually proved satisfactory and been all the remedy required to control the fever. Although I have never seen any alarming effects from its use, late in the disease, when the vital forces have become much weakened, I have not dared at all times to give doses sufficiently large to reduce the temperature, preferring, at that stage of the disease and condition of the patient, to give quinine, which came into use in larger doses at an earlier date. I have never known it to fail in reducing the temperature, when given in sufficiently large doses, but in this case did not find it free from danger.

To Mr. J. S., aged 28, ill three weeks with the fever, temperature 105 degrees, pulse 120, stomach, for the last week, had retained little food or medicine, I gave thirty grains sulphate of quinine, by the rectum, in acid solution with tablespoonful of brandy and four ounces of beef tea; the next morning, twelve hours later, the temperature had fallen to 96 2-5 degrees and did not rise to normal for seven days. During this time, the heart's action was so feeble that the patient could not be raised from the recumbent position without producing alarming syncope. Convalescence was established in ten days without any return of the fever, during which time, the patient took all food and medicine by the rectum.

It has always been my practice after the first week or whenever indicated, in order to guard against death by exhaustion, to sustain my patients by administering a good quantity of nourishment, generally milk; also to administer tonics, of which quinine to the extent of eight to sixteen grains per day has been my chief reliance. Alcoholic stimulants I have never neglected to use, whenever indicated by an approaching failure of the vital forces; and it is my belief that by the use of brandy or whisky, to the extent of six to ten ounces per day, I have been able to save the lives of patients in typhoid fever, who would, otherwise, have died. Antiseptics in some form I have used ever since I began practice. Why I commenced so early and what has been their effect, I do not even now know. At first I used a sulphate of soda; later, from the practice of my colleague, I came to rely upon the tincture of the chloride of iron, so on through the list of chemicals, until the past year, I have used only dilute nitromuriatic acid, and the result has been equally good. The condition of the alimentary canal I have never neglected, without regret. If the evacuations were not voluntary, formerly, I used castor oil; of late, I have used at first, a dose or two of mild chloride of mercury, followed by Seidlitz powder; later in the disease, Seidlitz powder only. For diarrhœa, I have usually relied upon opium and acetate of lead.

Intestinal hemorrhage and perforation, I have given as the third and probably most frequent cause of death. I have placed them together as the same cause, because they are the result of the same condition—and my success has been so uniform in their prevention that the motive which has prompted me to prepare this paper, has been to put on record my uniform success, which I attribute to the use of a single remedy, long known to all, but neglected by many. Probably, an average number of cases of fever occurring in a country practice have fallen to my lot, and of that number, I have never had a single case of intestinal hemorrhage or perforation. This I attribute to the judicious use of that old, neglected, and often despised remedy, oil of turpentine, which I have never failed to use whenever indicated. As soon as the tongue becomes dry, especially if the coating peels off, leaving it red and fissured, I commence the use of turpentine in eight-to-ten-drop doses, every four to six hours, given in emulsion or syrup, and continue its use until that signal of danger disappears. In many cases, I do not use it at all; but whenever indicated always. In my practice, I have never used any remedy with greater satisfaction or more immediate, uniform and favorable results. You may call it intestinal, antiseptic, or whatever you please; I care not what the theory is as long as the practice is a success. I commenced the practice of medicine with no text-book on practice except George B. Wood's, and in my limited experience, I have been more faithful in the use of this remedy introduced by him into the treatment of typhoid fever, than he himself was; and if others have used it as faithfully I feel assured it has served them equally as well.

That I have preserved the notes of only unusual and interesting cases and am, therefore, unable to give you the total number treated, is to me a source of regret. I have not seen an epidemic of the disease. Few cases only have appeared to contract it from others. My cases have been scattered through the town and adjoining towns, not more than twelve or fifteen in any one year, many years much less. Some years, no more than one or two, but the aggregate has been quite large. My only fatal cases have been an old lady eighty-two years of age, who died in the first week without a positive diagnosis, and a man, sixty-eight years old, financially and physically broken down, two of whose brothers had committed suicide, who, after passing through the fever stage, refused medicine and food because he did not wish to live, and died of asthenia, near the end of the fourth week. With these exceptions, my patients have all lived, and excepting the impaired lung in the case of abcess, all have been restored to full health.

Typhoid fever is a formidable disease, to me, often fraught with the greatest danger; nevertheless, my results have been so favorable that when death claims my patients, I shall feel that there has been want of due care on the part of the patient, the attendant or myself.

---

THIRTEEN FEET OF INTESTINES TORN AWAY.—A few weeks ago I was called to see a lady who had either performed an abortion on herself or some one had done so for her. The fœtus had been delivered, but the placenta remained in the cavity of the uterus. A physician was called, who discovered something protruding from the os, and, upon investigation, found it was an intestine. There was considerable hemorrhage and very severe pain, with the prospect of speedy death. I found the pulse and respiration very rapid and a profuse hemorrhage from the vagina. As soon as possible I opened the abdomen, and found the uterus about the size of a five months' pregnancy and very soft, the abdominal cavity quite filled with blood. Upon lifting the uterus out I discovered, on its posterior surface, an opening, through which passed a loop of small intestine. This I immediately withrew; its mesentery had been stripped entirely off. One end of the intestine had been torn from its attachment to the cæcum. I inserted my finger into the cavity of the uterus, and, finding the placenta, delivered it, enlarging the opening. I cut off the intestine at the point where the mesentery stopped, making a lateral anastomosis with the cæcum on the opposite side. I then closed the wound. Thirteen feet of the intestine had been drawn through the opening in the uterus. The patient died at 12:30 A. M., one remarkable feature being that she survived the injury and the subsequent operative procedure over eight hours.—DR. C. B. NICHOLS, in *Occidental Medical Times*.

